# Marker Gene-Guided Graph Neural Networks for Enhanced Spatial Transcriptomics Clustering

**DOI:** 10.53941/aim.2025.100001

**Published:** 2025-02-07

**Authors:** Haoran Liu, Xiang Lin, Zhi Wei

**Affiliations:** 1 Department of Computer Science, New Jersey Institute of Technology, Newark, NJ 07102, USA; 2 Department of Biomedical Informatics, Harvard Medical School, Boston, MA 02115, USA

**Keywords:** spatial transcriptomics (ST), marker gene, graph neural network (GNN), contrastive learning

## Abstract

Recent advancements in Spatial Transcriptomics (ST) technologies have enabled researchers to investigate the relationships between cells while simultaneously considering their spatial locations within tissue. These technologies facilitate the integration of gene expression data with spatial information for clustering analysis. While many clustering methods have been developed, they typically rely on the dataset’s intrinsic features without incorporating domain knowledge, such as marker genes. We argue that incorporating marker gene information can enhance the learning of cell embedding and improve clustering outcomes. In this paper, we introduce MGGNN (Marker Gene-Guided Graph Neural Networks), a novel approach designed to enhance spatial transcriptomics clustering. Firstly, we train the model using a contrastive learning framework based on a Graph Neural Network (GNN). Subsequently, we fine-tune the model using a few spots labeled by the expression of marker genes. Simulation and experiments conducted on two real-world datasets demonstrate the superior performance of our model over state-of-the-art methods.

## 1. Introduction

Advances in spatial transcriptomics (ST) enable scientists to explore the relationships between cells while preserving their spatially resolved gene expression within the original tissue section [1, 2]. Furthermore, recent studies [3–5] have demonstrated that spatial organization, particularly in the brain, is fundamentally related to its function. Therefore, analyzing and understanding these spatially resolved gene expressions is crucial for pathology [6, 7].

Due to the high dimensionality of the original data, most methods apply dimensionality reduction before conducting downstream analyses. The goal of dimensionality reduction is to learn a suitable representation (embedding) of the original data in a lower-dimensional space, typically referred to as the latent or feature space. After obtaining these representations, downstream analyses such as clustering can be performed. In the field of single-cell RNA (scRNA) data analysis, popular tools such as Seurat [8] have adapted Principal Component Analysis (PCA) for this purpose. The goal of PCA is to extract valuable components from the original data, known as principal components (PCs), which are then used for downstream analyses. Although PCA-based methods are widely adopted, the downstream analysis tasks are often isolated from the dimensionality reduction process. This is because the representation learning is not specifically designed for downstream tasks such as clustering, which can limit overall performance. Furthermore, PCA-based methods or other machine learning based approaches such as AL model [9] and scDCC [10] do not consider spatial information during the dimensionality reduction process, meaning that the mapping from the original data space to the embedding space does not benefit from spatial context.

Recently developed methods [11–17] have attempted to incorporate spatial information into their models, leading to improved clustering performance. For example, BayesSpace employs a statistical approach that utilizes information from spatial neighborhoods. Additionally, many newer deep learning methods are based on graph neural network (GNN) models. Since the collected spots are well distributed in the tissue sections, a graph can be intuitively constructed based on spatial coordinates, with the spots treated as nodes, gene expressions as node features, and edges formed between nearest neighbors. Additionally, SpaGCN connects spots with similar gene expression profiles, while spaVAE employs a deep generative spatial variational autoencoder model.

Moreover, some researchers have introduced the concept of contrastive learning [18] into their models. The motivation behind contrastive learning is to leverage the information provided by the data itself to learn better representations for downstream tasks, especially since labeled data can be costly and difficult to obtain. Contrastive learning is typically implemented by defining positive and negative pairs among sample augmentations. The underlying intuition is straightforward: by pulling together positive pairs of samples augmentations while separating negative pairs in the embedding space, the embeddings learned by the model should be more meaningful, thereby enhancing the performance of downstream analyses. Consequently, the definitions of positive and negative pairs are critical. In the context of spatial transcriptomics data, positive pairs can be defined as augmentations from the same spot, whereas negative pairs are augmentations from different spots [11]. When considering the graph structure, negative pairs are typically limited to neighboring spots rather than spanning the entire graph. Recent studies have proven that this technique can enhance model performance in the context of ST data [19, 20].

Although state-of-the-art methods have adopted various technologies to enhance performance, none have effectively integrated domain knowledge into their models. Domain knowledge provides rich, data-related information that can significantly improve analysis. For example, during the annotation process, scientists can assign labels to clusters generated by analysis tools with the help of marker genes. Intuitively, one would expect that cells with similar marker gene expression tend to cluster together. However, the unsupervised learning-based methods mentioned above are not designed to accommodate domain knowledge.

We argue that by incorporating domain knowledge, such as marker genes, into the model, the embedding process can be constrained and directed towards more meaningful clustering results. We also note that some scRNA-seq clustering methods, such as CellAssign [21], scDCC [10], have successfully utilized domain knowledge to enhance clustering performance. However, ST data exhibits strong spatial dependencies; that is, spatially close spots typically belong to the same cell type, which imposes additional constraints on the model when using domain knowledge. For instance, in scRNA-seq analysis, cells with similar domain knowledge observations (such as marker gene expression) can be considered as belonging to the same cluster, since the actual spatial distances between them are not available and thus impose no restrictions. In contrast, for ST data, spots with similar marker gene expression that are spatially distant from the main group of spots may be outliers. Furthermore, domain knowledge is often only partially aligned with the dataset, which means that an effective ST data-based model must also manage the noise introduced by this domain knowledge. While some methods, such as DSSC [16] and SPAN [22], utilize both marker gene and spatial information, they do not clearly demonstrate their models’ robustness against misinformation. This distinguishes MGGNN, which explicitly addresses and performs well under such conditions.

In this study, we developed a marker gene-based spatial transcriptomics clustering method, MGGNN, to address key challenges in the field:

- Limited Integration of Domain Knowledge: Existing methods often fail to incorporate domain knowledge, such as marker genes, into clustering workflows.
- Lack of Robustness Against Noise: Many approaches are sensitive to inaccuracies in domain knowledge, limiting their applicability to real-world data.

The MGGNN solutions are:

- Enhanced Biological Relevance: MGGNN leverages marker genes during both the embedding and clustering stages, effectively integrating domain knowledge to improve clustering outcomes.
- Proven Robustness Against Noise: MGGNN demonstrates resilience to noisy marker gene-identified labels, as validated through extensive simulations and experiments on real-world datasets.

To effectively integrate spatial information with gene expression data, MGGNN employs a self-supervised graph neural network (GNN) during pre-training. Specific spot labels, identified based on marker gene expression, guide the fine-tuning stage to enhance clustering accuracy. Experiments on simulations and two real-world datasets validate MGGNN’s robustness to noise and its superior performance compared to state-of-the-art methods.

## 2. Materials and Methods

### 2.1. DLPFC Dataset

This dataset comprises 12 human dorsolateral prefrontal cortex (DLPFC) samples, which were annotated and presented by Maynard et al. [4] based on the cytoarchitecture and markers genes such as SNAP25, PCP4, and MOBP. Each sample was divided into cortex layers and white matter (WM), and the dataset can be accessed through the spatialLIBD package [23].

### 2.2. Coronal Mouse Brain Dataset

This dataset was presented by Akeret et al. [24] and comprises 7 samples. The original dataset was acquired after striatal injection of hemoglobin and heme-hemopexin. Each sample was manually segmented into 7 distinct anatomical structures.

### 2.3. Simulation

To evaluate the performance and robustness of our model, we conduct simulations. Practically, domain knowledge, such as marker genes, can vary across datasets and may introduce noise depending on specific situations. Therefore, we designed and generated a simulation dataset that intentionally controls the amount of information provided by marker genes to the model and the quality of that information. As previously mentioned, our model initially identified a few spots and then utilized them to benefit the rest of the model. We randomly selected *p* percentage (e.g., 15% when *p* = 0.15) of these spots and intentionally masked *q* percentage of these labels by assigning them false labels, while the remaining labels retained their true labels. Together, these labels are considered identified by domain knowledge and revealed to the model. Specifically, the value of *p* is selected from {0.15, 0.2, 0.25, 0.3}, and the value of *q* is selected from {0.1, 0.2, 0.3, 0.4, 0.5}. For each pair of {*p*, *q*}, we repeat the process 10 times based on the sample 151673 of the DLPFC dataset and then test methods on the remaining 1 − *p* percentages of spots. The performance of each method is presented in the results section. The reason we chose sample 151673 is that this sample serves as a representative demonstration of the structure of the widely used DLPFC dataset and has been used as an example in the original paper.

### 2.4. Overview of MGGNN

**Figure 1.**
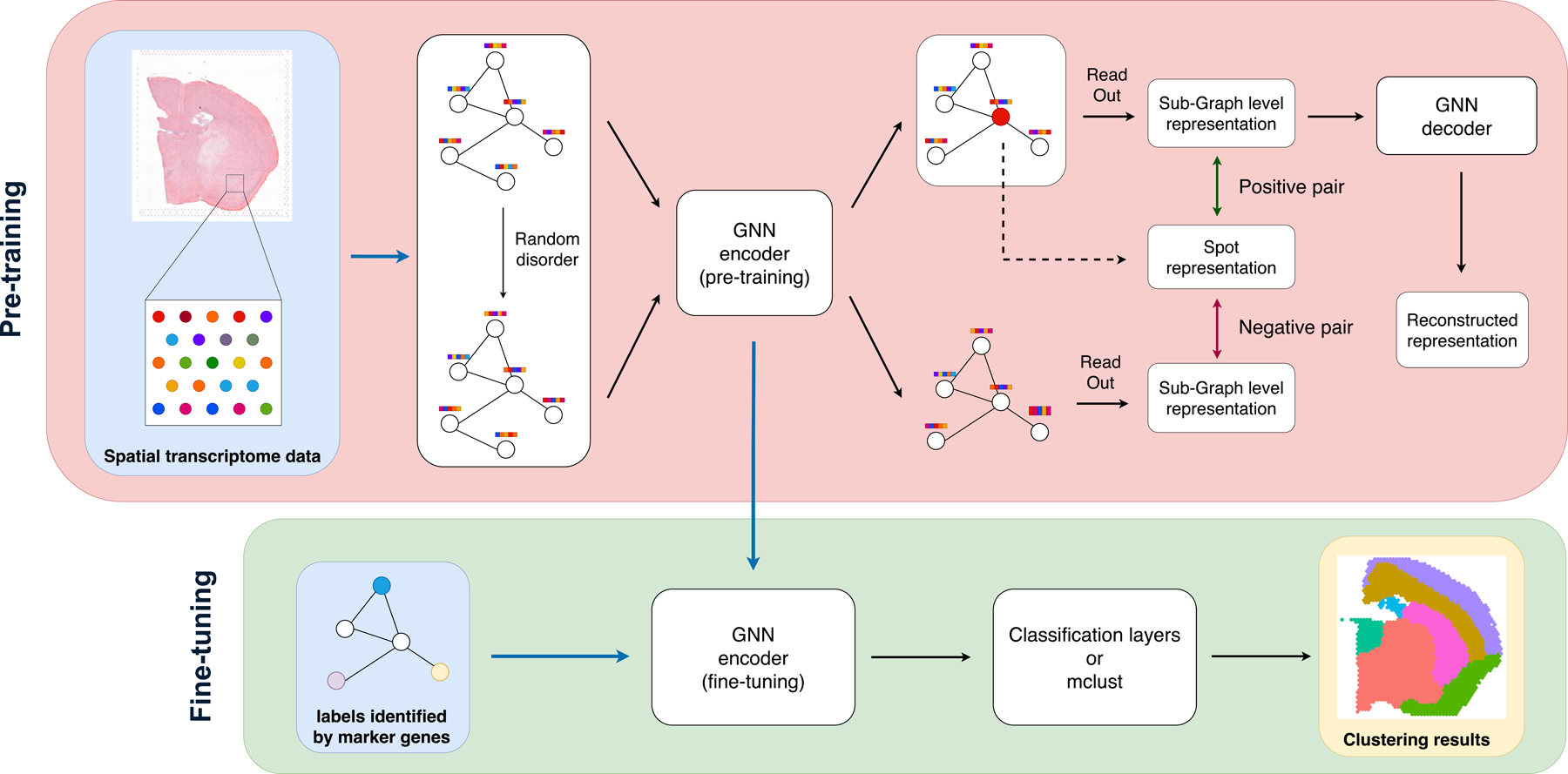
MGGNN Framework. The MGGNN model accepts ST data and a few labeled spots as input. The labeled spots can be user-provided or identified using marker genes. During pre-training, contrastive learning helps the model output meaningful embeddings, while fine-tuning uses domain knowledge-identified labels to guide clustering. MGGNN supports two clustering methods: classification layers for high-quality labels with strong spatial dependence, or an unsupervised approach (mclust) for embedding-based clustering when label quality is uncertain.

The framework of MGGNN can be divided into two distinct phases as shown in Figure 1: Pre-training and Fine-tuning. Before training the model, we identify labels for a limited number of samples to use in the fine-tuning phase. In this study, the labels were identified using marker genes, but our model is adaptable to any domain knowledge identified label. If the user has their own preferred identified spots, this step can be omitted.

For the model pre-training phase, we primarily follow the workflow of GraphST, the coordinates of spots are used to construct a graph, while the normalized gene expression is treated as the node features of the graph. Then, we randomly shuffle the normalized gene expression of spots to generate a new graph for the subsequent contrastive learning. These graphs are fed to the same GNN encoder. For each spot, we extract its nearest neighbors to form a sub-graph. We set the sub-graph level representation sampled from the original graph and the spot gene expression as the positive pair, while the sub-graph level representation sampled from the generated graph and the spot gene expression as the negative pair. The contrastive learning loss minimizes the distance between the positive pairs while maximizing the distance between the negative pairs. We also introduce a GNN decoder following the encoder to reconstruct the normalized gene expression.

For the fine-tuning step, we introduce a few marker-gene-identified spot labels to the encoder to train a classifier that correctly predicts these spots’ labels. Finally, based on the percentage of the identified spots over all spots, we dynamically use the classifier or an unsupervised learning algorithm to cluster the spots.

Detailed explanations are provided in the following subsections.

### 2.5. Label Identification by Marker Genes

MGGNN requires a list of marker genes for each cell type to identify the labels of a few spots. The number of unique marker genes for each cell type must be greater than one. Practically, if users can provide labels identified through other domain knowledge, the marker genes are not necessary for the model. The process of label identification is based on two intuitive assumptions: (1) Spots with higher marker gene expression are more likely to belong to the corresponding cell type; (2) Spots belonging to the same cell type tend to be spatial neighbors and thus may exhibit similar gene expression patterns. Once marker genes are provided, the model first filters out unique marker genes for each cluster. Next, Seurat’s sctransform [25] function is used to normalize the data, followed by the computation of the top 15 principal components (PCs). For each filtered marker gene, spots are ranked based on normalized expression values, from high to low. For clusters with more than one marker gene, the ranks of each spot are summed, and this cumulative rank is used as the ranking criterion for spots.

Starting with the top-ranked spot, labels are assigned by applying restrictions based on the aforementioned assumptions. The procedure is as follows: (1) Identify the top-ranked spot as belonging to the corresponding cell type. (2) Evaluate the next-ranked spot and compute the mean expression of its nearest neighbors. (3) Compare the mean expression of the 30 PCs for this spot’s neighbors with that of the previously ranked spot’s neighbors. If the cosine similarity between these two sets of mean expressions exceeds a certain threshold (0.05), the spot is retained and assigned the same label. If not, a “patient value” is incremented. This process continues until the patient value exceeds a pre-set threshold, or the total number of identified spots surpasses a defined limit. This limitation prevents over-identification of clusters with a small number of spots.

### 2.6. Data Preprocessing

MGGNN processes three main types of input data: Gene expression, spatial coordinates, and a few marker gene-identified spots. As previously mentioned, users have the flexibility to provide spots identified using other domain knowledge, allowing for adaptability in various research contexts. For the gene expression data, raw counts are first log-transformed and normalized using the Scanpy [26] package in Python. Following normalization, the top 3000 highly variable genes (HVGs) are selected. These HVGs are then utilized in the subsequent stages of model pre-training and fine-tuning.

### 2.7. Grpah Construction

The graph structure forms the foundation of the GCN model and is critical to its performance. Since the spots are evenly distributed across the slices, we intuitively connect each spot with its nearest neighbors. The graph can be represented as $G=\left(V,E\right)$, where V denotes the nodes of the graph and E represents the edges. In the case of ST data, each spot is treated as a node, and edges are constructed by connecting each spot to its three nearest neighbors based on Euclidean distance. This approach is appropriate because the spots collected from the 10× platform are evenly distributed on the slice. For each spot (excluding those on the edges of the sample), there are typically three neighbors with the same shortest distance to the spot. The graph is then converted into an adjacency matrix A for easier processing by the model. This adjacency matrix is defined as $A\in {\mathbb{R}}^{N_{\text{spot}}\times N_{\text{spot}}}$, where $a_{ij}=1$ if there is an edge between nodes i and j, and $a_{ij}=0$ otherwise.

### 2.8. GCN Structure

The GCN encoder in MGGNN consists of multiple Graph Convolutional Network (GCN) [27] layers. Each layer processes the graph’s structural information and spot features to extract meaningful representations. The operation of a single GCN layer is defined as:

(1) $$H^{\left(l+1\right)}=\sigma \left({\tilde{D}}^{-\frac{1}{2}}\tilde{A}{\tilde{D}}^{-\frac{1}{2}}H^{\left(l\right)}W^{\left(l\right)}+b^{l}\right)$$

where:

- $\tilde{A}=A+I_{N}$ is the adjacency matrix of the graph G with added self-connections ($I_{N}$ is the identity matrix).
- $\tilde{D}$ is the degree matrix of $\tilde{A}$.
- $H^{\left(l\right)}\in {\mathbb{R}}^{N\times D}$ is the output of the l-th layer, initialized as $H^{\left(0\right)}=X$, where X is the input node feature matrix.
- $W^{\left(l\right)}$ is a trainable weight matrix, and $b^{l}$ is the bias vector for the l-th layer.
- $\sigma \left(\cdot \right)$ denotes a non-linear activation function, such as ReLU.

This formulation ensures that information is aggregated from neighboring nodes and propagated through the graph in a weighted manner, capturing both local and global graph structures.

The GCN decoder mirrors the encoder structure and aims to reconstruct the original gene expression matrix $\left(\tilde{X}\right)$. The reconstruction loss is calculated as:

(2) $$L_{\text{recon}}=\sum_{i=1}^{N_{\text{spot}}}\|x_{i}-{\tilde{x}}_{i}{\|}_{F}^{2}$$

This loss ensures that the embeddings learned by the GCN encoder retain the critical information necessary for reconstructing the input data, preserving biological relevance.

### 2.9. Graph-Based Contrastive Learning

Graph-based contrastive learning is a key component of MGGNN, designed to extract meaningful representations from unlabeled ST data. It achieves this by optimizing embeddings to distinguish between similar (positive) and dissimilar (negative) data pairs, which are generated through data augmentation.

Data augmentation is a crucial step in contrastive learning, as it produces the positive and negative pairs needed for subsequent training. For ST data, the node features represent the gene expression of each spot. Traditional data augmentation techniques commonly used for image data, such as cropping and color distortion, are not meaningful in this context. A straightforward approach involves masking parts of the gene expression data. However, simple masking fails to fully utilize the rich information embedded in the graph structure.

Positive pairs are created by utilizing spatial relationships within the graph. Specifically, the embedding representation of a spot and the readout of a subgraph (comprising the spot itself and its neighbors) form a positive pair. The readout function is defined as the sigmoid of the mean of the representations of the immediate neighbors. This strategy ensures that the embeddings capture both local structure and gene expression information.

In contrast, negative pairs are generated by introducing randomness into the data. For example, normalized gene expression in the augmented graph is randomly reordered, disrupting spatial and expression patterns. The readout of these augmented subgraphs is unlikely to relate closely to the original spots, ensuring a meaningful distinction from positive pairs.

The loss function for positive pairs is defined as:

(3) $$L_{SCL}=-\frac{1}{2N_{\text{spot}}}\left(\sum_{i=1}^{N_{\text{spot}}}\left(E_{\left(X,A\right)}\left[\log\text{Φ}\left(z_{i},g_{i}\right)\right]+E_{\left(X^{\prime },A^{\prime }\right)}\left[\log\left(1-\text{Φ}\left(z_{i}^{\prime },g_{i}\right)\right)\right]\right)\right)$$

where:

- $z_{i}$ and $g_{i}$ represent the embedding of a spot and the readout of its subgraph, respectively.
- X and A denote the original feature and adjacency matrices, while $X^{\prime }$ and $A^{\prime }$ are their augmented counterparts.
- $\Phi \left(\cdot \right)$ is a similarity function, typically a dot product or cosine similarity.

The loss ensures that embeddings of positive pairs are drawn closer together, while embeddings of negative pairs are pushed apart.

Similarly, a corresponding loss function for negative pairs, $L_{SCL\text{\_}corrupt}$, is defined to reinforce this separation.

The overall pre-training loss combines the reconstruction loss from the GCN decoder and the contrastive losses:

(4) $$L=\lambda_{1}\;L_{recon}+\lambda_{2}\left(L_{SCL}+L_{SCL\_corrupt}\right)$$

where $\lambda_{1}$ and $\lambda_{2}$ are scaling factors that balance the importance of reconstruction and contrastive learning. In this study, these values are empirically set to 10 and 1, based on the established settings in GraphST, which have been proven effective in related studies.

By aligning embeddings with both structural and feature-level relationships, graph-based contrastive learning ensures that the representations learned by MGGNN are robust and biologically meaningful. This step is critical for downstream clustering and annotation tasks, as it captures both spatial and molecular patterns inherent in the ST data.

### 2.10. Fine-Tuning

After completing the pre-training stage, the model transitions to fine-tuning. In this stage, the weights learned during pre-training are retained as the initial weights, rather than being randomly initialized. This strategy leverages the pre-trained features for better downstream performance. Using marker gene-identified labels, we integrate additional Multi-Layer Perceptrons (MLPs) that process the encoder’s output for the task of label classification. Given the typically small number of labeled spots (on the order of hundreds), an early stopping mechanism is applied during fine-tuning to address two key challenges: (1) Mitigating Overfitting: Due to the small number of labeled spots, the loss function decreases rapidly during the initial stages of classification. Prolonged training risks overfitting the labeled spots, reducing the model’s generalization capability across all spots. (2) Handling Noisy Labels: The labels are derived from marker genes, which may inherently include noise. Therefore, it is unnecessary for the model to perfectly classify every labeled spot. The fine-tuning process is halted when the classification accuracy (evaluated using marker gene-identified labels rather than ground truth labels) exceeds 0.9. Once fine-tuning is complete, clustering or classification methods are applied to the learned embeddings. The approach depends on the proportion of labeled spots: (1) If labeled spots are lesser than 20% of total spots, the dataset is clustered using the mclust [28] algorithm, as the classifier trained on a small number of labeled spots may overfit and fail to generalize. (2) If labeled spots are greater than 20% of total spots, the classifier trained during fine-tuning is applied to classify the entire dataset. The increased number of labeled spots ensures that the classifier is less prone to overfitting, indicating that the marker gene expressions sufficiently represent the dataset.

## 3. Results

Our experiments demonstrate that MGGNN produces results consistent with domain knowledge. MGGNN ensures that domain knowledge directly contributes to clustering or classification, resulting in outcomes that are both meaningful and aligned with biological insights. MGGNN is flexible, allowing users to customize their analysis by providing predefined spots’ labels if desired. This flexibility, combined with its integrated approach, ensures MGGNN is a robust tool for spatial transcriptomics analysis.

### 3.1. Simulation Experiments and Performance Evaluation

**Figure 2.**
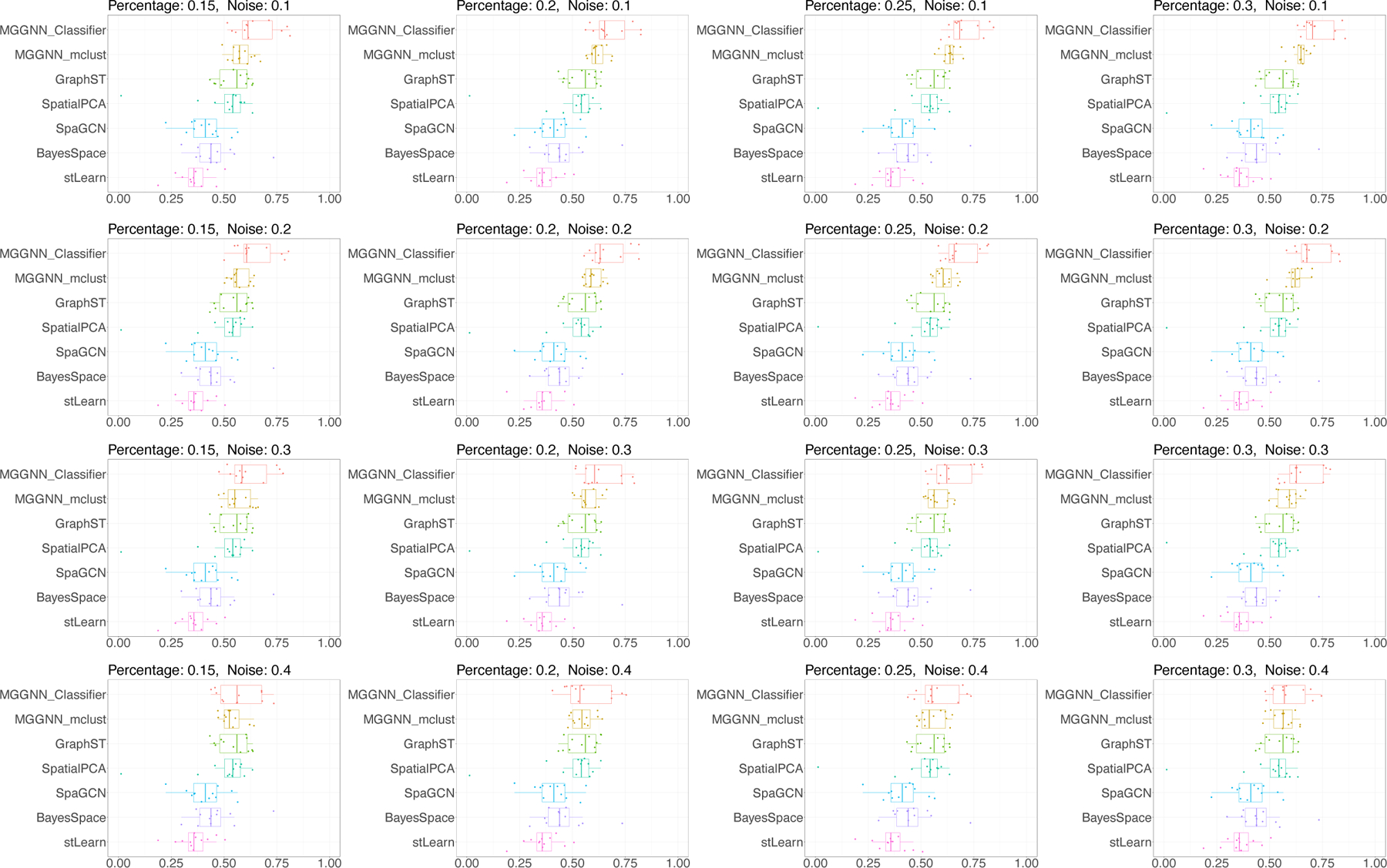
Simulation Results. In each simulation, we sampled *p* percentage of spots and introduced *q* percentage noise by assigning incorrect labels during fine-tuning. MGGNN outperformed state-of-the-art methods under noise levels below 40% and maintained competitive performance even at 40% noise.

We evaluated MGGNN using simulations with varying levels of noise and sampling percentages. The Adjusted Rand Index (ARI) was used to measure clustering performance, with higher ARI values indicating closer alignment with manual annotations. MGGNN was the only method provided with additional information under different noise levels, and its performance was compared to state-of-the-art methods as shown in Figure 2.

When the noise level was low (*q* = 0.1), MGGNN outperformed all other methods. It maintained a leading position when the noise level was no more than 0.3. At higher sampling percentages *p*, MGGNN demonstrated improved performance due to the increased availability of marker gene information. Notably, even at a noise level of 0.4—where only 60% of the sampling labels were correct—MGGNN achieved competitive performance comparable to GraphST when *p* = 0.3, highlighting its robustness against noise.

Further analysis revealed that MGGNN_Classifier outperformed MGGNN_mclust at noise levels below 0.3. However, as noise increased, MGGNN_Classifier became more sensitive, whereas MGGNN_mclust showed greater resilience.

In practice, when the number of spots is approximately 5,000 (with 20% equating to 1,000 spots), deep learning-based classifiers may overfit. To address this, MGGNN dynamically switches between MGGNN_Classifier and MGGNN_mclust based on the percentage of marker gene-identified labels. When the percentage exceeds 20%, MGGNN utilizes MGGNN_Classifier. Conversely, for percentages below 20%, it defaults to MGGNN_mclust to prevent overfitting and ensure robustness in smaller datasets.

### 3.2. Performance on Human Dorsolateral Prefrontal Cortex (DLPFC) Dataset

**Figure 3.**
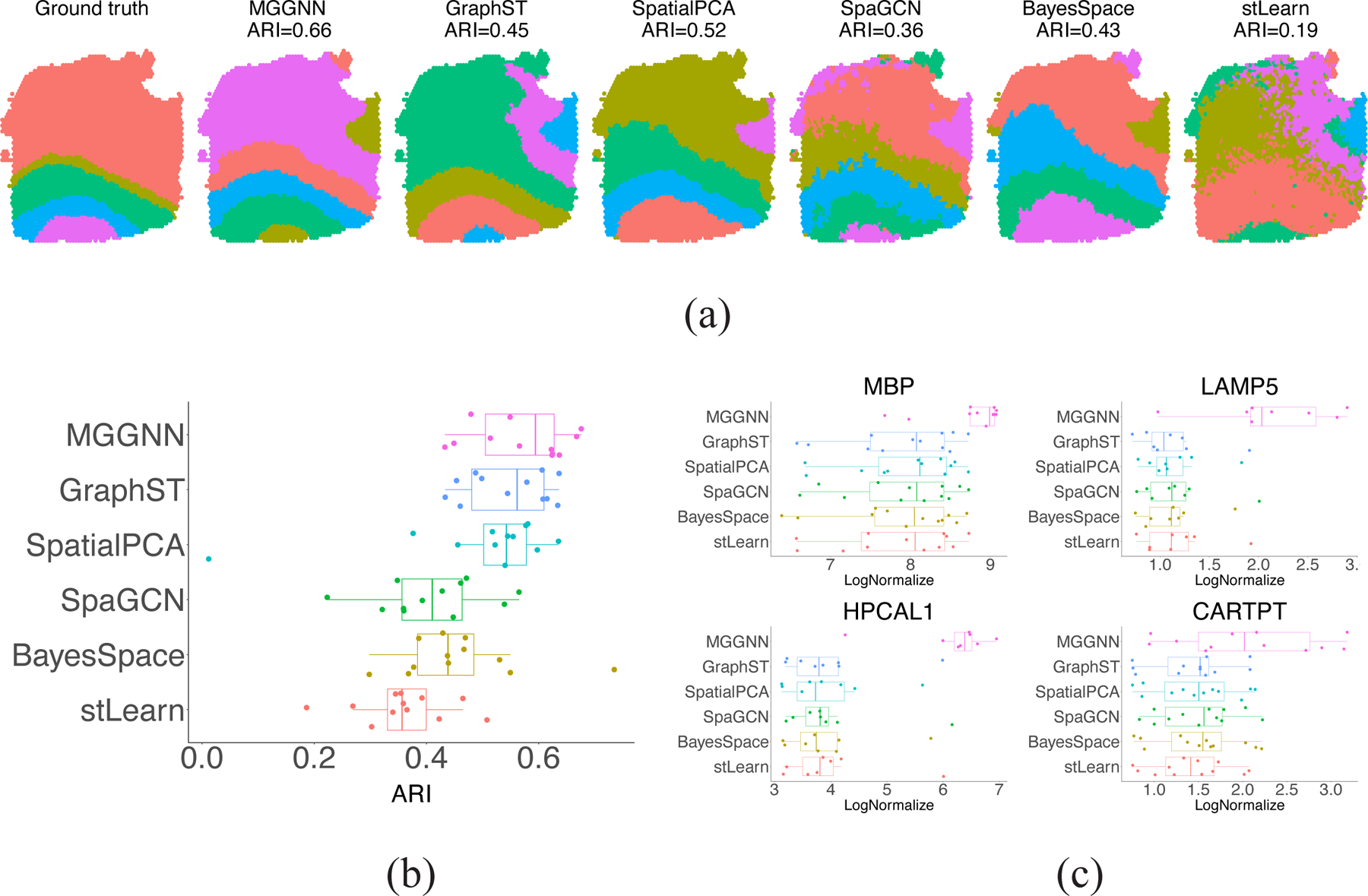
Performance on DLPFC Dataset. (**a**) Clustering results for sample 151670, where MGGNN closely matches the ground truth. (**b**) Overall performance across 12 DLPFC samples, highlighting MGGNN’s competitiveness. (**c**) Marker gene expression levels in corresponding layers across 12 samples, showcasing MGGNN’s superior alignment with domain knowledge.

MGGNN’s performance was validated on the DLPFC dataset, which consists of 12 samples annotated with spatially resolved gene expression data. As shown in Figure 3a, MGGNN produced clustering results for sample 151670 that most closely resembled the ground truth, effectively capturing the spatial structure of the dataset.

Figure 3b compares the performance of various methods across all samples, demonstrating MGGNN’s consistent competition to other methods, a quantitative demonstration is detailed in Supplementary Table S1. Although GraphST achieved similar ARI values, MGGNN’s clustering results aligned more closely with domain knowledge, as highlighted in Figure 3c. Each dot represents the average marker gene expression in the corresponding layer of one sample. Since there are 12 samples, there are 12 dots for each method. For example, the marker gene MBP, specific to white matter (WM), showed concentrated expression in WM clusters identified by MGGNN. MGGNN achieved the highest expression levels across all 18 marker genes (detailed in Supplementary Figure S1) used to identify spots, further highlighting its capability to effectively integrate domain knowledge.

### 3.3. Performance on Coronal Mouse Brain Dataset

**Figure 4.**
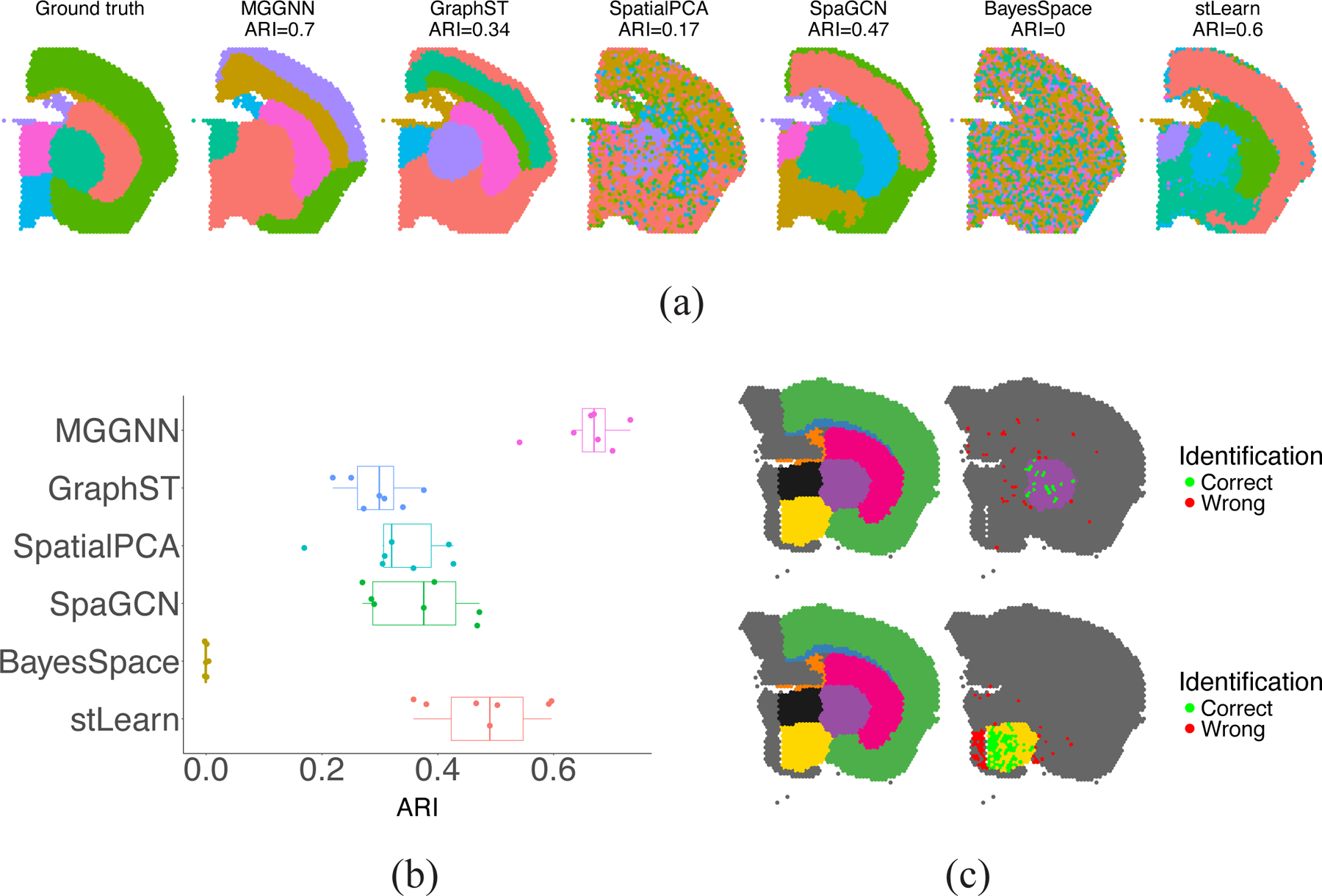
Performance on Coronal Mouse Brain Dataset. (**a**) Clustering results for a sample treated with 10 nmol of heme, where MGGNN achieves the highest ARI and closely matches the ground truth. (**b**) Performance comparison across all samples, highlighting MGGNN’s superior ARI. (**c**) Marker genes identified spots (green: correct, red: incorrect), demonstrating MGGNN’s robustness in leveraging domain knowledge.

The coronal mouse brain dataset was used to further evaluate MGGNN. Despite carefully following the instructions for BayesSpace, it failed to produce meaningful results, possibly due to issues in handling input data. Compared to other methods, MGGNN demonstrated a clear advantage by achieving the highest ARI scores for a sample treated with 10 nmol of heme and producing clustering results most similar to the ground truth (Figure 4a).

Figure 4b illustrates MGGNN’s superior clustering accuracy across all samples in this dataset, benefiting from the inclusion of marker gene-identified spots (a quantitative demonstration is detailed in Supplementary Table S2). Figure 4c further highlights MGGNN’s robustness by visualizing identified spots of the control sample, with correct and incorrect identifications marked in green and red, respectively. The left side shows the ground truth, while the right side displays the identification of a specific layer. Even in cases of partial misidentifications, MGGNN consistently outperformed other methods, demonstrating its resilience and effective utilization of domain knowledge.

## 4. Discussion

In this study, we proposed MGGNN, a novel framework for spatial transcriptomics clustering that integrates marker gene-based embedding into the main analytical workflow. The effectiveness of MGGNN was demonstrated through experiments that produced results consistent with domain knowledge, validating its reliability in practical applications. Marker genes, typically limited to the annotation stage in conventional workflows, play a broader role in MGGNN by informing the embedding and clustering stages. This integration allows the model to produce results that are more aligned with domain knowledge while also regularizing clustering outcomes. The flexibility of MGGNN is another key advantage. Users can customize the model by providing their own labeled spots, offering adaptability to diverse datasets and research goals. Moreover, the self-supervised pre-training framework allows MGGNN to maximize the utility of the raw data. Our study demonstrates that even with a small number of identified spots, MGGNN can achieve meaningful clustering and maintain robustness against potential noise. On the other hand, unlike methods such as SpaGCN, which calculate edge weights based on pre-defined metrics like physical distance or histological information, MGGNN learns edge weights during training using attention mechanisms. This approach reduces reliance on prior assumptions, making the model simpler and more versatile.

Like any advanced method, MGGNN has certain limitations. A key dependency is the availability and quality of layer-specific marker genes. In datasets where marker gene knowledge is limited, incomplete, or inaccurate, MGGNN’s performance may be adversely affected, as it relies on these genes to guide clustering and embedding.

Another limitation is the trade-off between flexibility and interpretability. MGGNN dynamically learns edge weights, enabling it to adapt to diverse datasets. However, this flexibility introduces additional complexity, making it more challenging to interpret the underlying graph structures compared to models with explicitly defined and fixed edge weights.

Future work could focus on reducing the dependency on marker genes by incorporating additional sources of domain knowledge or developing methods to infer pseudo-marker genes from the data itself. Expanding the model to include multi-modal data, such as histological images, could further enhance its robustness and applicability.

## Supplementary Material

aim.2025.100001-Supplementary Materials

The following supporting information can be downloaded at: https://www.sciltp.com/journals/aim/2025/1/654/s1, Figure S1: Marker Gene Expression. Marker gene expression levels across 12 samples in corresponding layers demonstrate MGGNN’s superior alignment with domain knowledge. All marker genes used for spot identification are listed; Table S1: The ARI results for each sample from the DLPFC dataset; Table S2: The ARI results for each sample from the Coronal Mouse Brain dataset.

## Data Availability

The datasets analyzed in this paper are available via links provided in the original papers. The code related to our work is available at https://github.com/Haoran-Liu/MGGNN, accessed on 1 January 2025.
