# Supplementary material for: Marker Gene-Guided Graph Neural Networks for Enhanced Spatial Transcriptomics Clustering
Source: AI Med. Author manuscript; Available in PMC 2025 Sep 5. (PMC12410142; doi:10.53941/aim.2025.100001)
Supplement: aim.2025.100001-Supplementary Materials [file NIHMS2064897-supplement-aim_2025_100001-Supplementary_Materials.pdf]

Scilight

AI Medicine
https://www.sciltp.com/journals/aim

Supplementary Information: Marker Gene-Guided Graph Neural Networks for Enhanced Spatial Transcriptomics Clustering

Haoran Liu [1], Xiang Lin [2] and Zhi Wei [1,*]

[1] Department of Computer Science, New Jersey Institute of Technology, Newark, NJ 07102, USA
[2] Department of Biomedical Informatics, Harvard Medical School, Boston, MA 02115, USA
* Correspondence: zhiwei@njit.edu

.

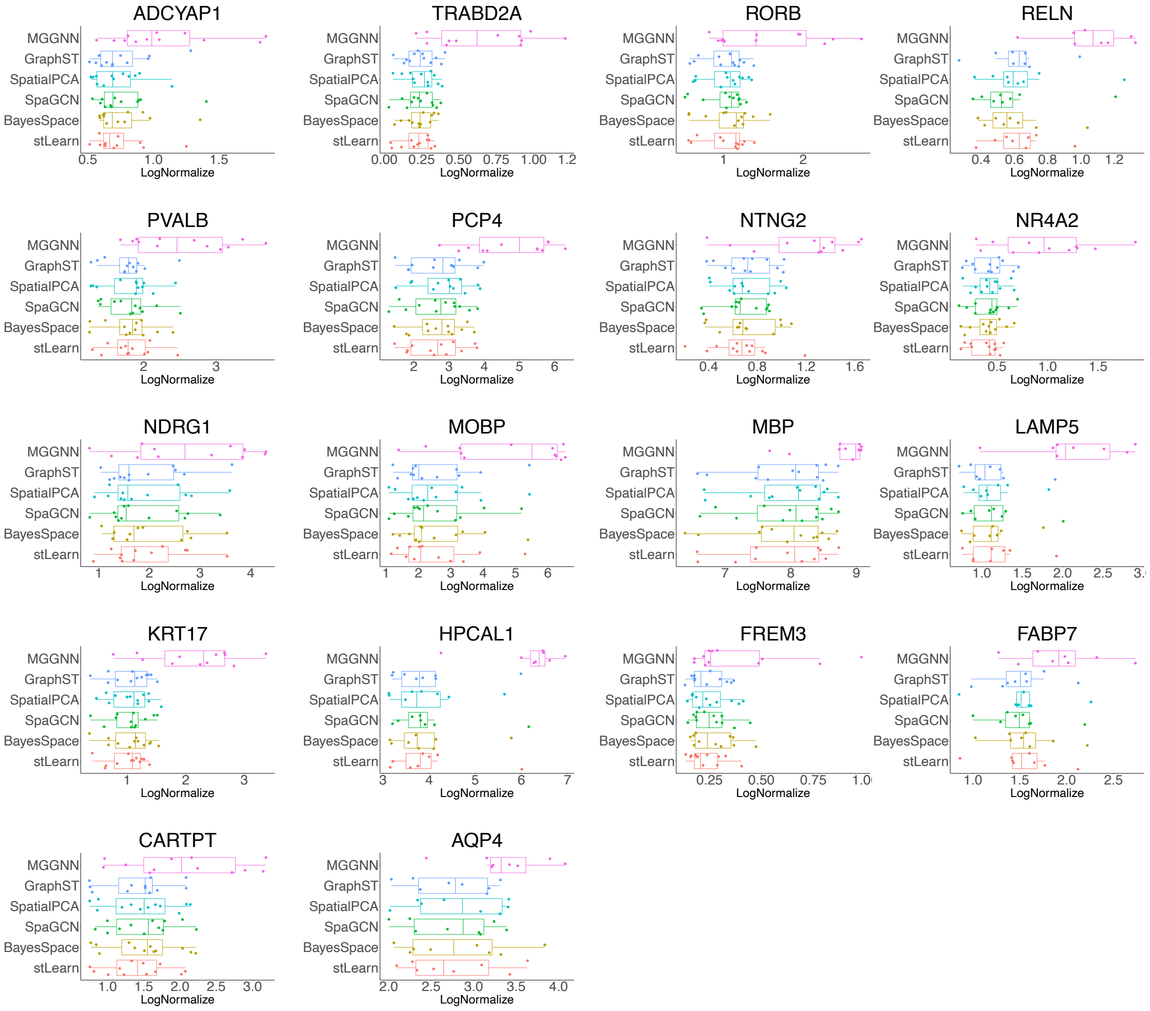

Figure S1. Marker Gene Expression. Marker gene expression levels across 12 samples in corresponding layers demonstrate MGGNN's superior alignment with domain knowledge. All marker genes used for spot identification are listed.

Marker gene expression levels across 12 samples in their corresponding layers demonstrate MGGNN's superior alignment with domain knowledge. Figure 1 shows all the marker genes used for spot identification. MGGNN successfully outputs results aligned with domain knowledge, as the expression of marker genes is predominantly observed in their corresponding layers.

Copyright: © 2025 by the authors. Submitted for possible open access publication under the terms and conditions of the Creative Commons Attribution (CC BY) license (https://creativecommons.org/licenses/by/4.0/).
Publisher's Note: Scilight stays neutral with regard to jurisdictional claims in published maps and institutional affiliations.

Table S1. The ARI results for each sample from the DLPFC dataset.

Sample	MGGNN	GraphST	SpatialPCA	SpaGCN	BayesSpace	stLearn
151507	**0.55846**	0.43335	0.53999	0.47120	0.46682	0.46493
151508	**0.52695**	0.49854	0.51746	0.35908	0.43740	0.30237
151509	**0.51714**	0.46005	0.45578	0.46106	0.38636	0.42265
151510	**0.50144**	0.48753	0.01190	0.42801	0.37719	0.26876
151669	**0.62299**	0.60749	0.37609	0.22305	0.46921	0.33993
151670	**0.60254**	0.45355	0.52229	0.35971	0.42905	0.18632
151671	0.59812	0.60601	0.59746	0.53876	**0.73454**	0.50777
151672	0.60818	**0.61470**	0.58062	0.56495	0.43897	0.34483
151673	**0.64926**	0.63622	0.57701	0.44761	0.54944	0.36521
151674	0.52026	**0.57885**	0.55435	0.39252	0.29805	0.35454
151674	0.50656	0.54447	**0.54468**	0.34806	0.53023	0.39194
151676	0.62310	0.63385	**0.63523**	0.32119	0.36791	0.35866

This is a quantitative demonstration of Figure 3b. The highest ARI values are listed in bold in the table. MGGNN achieved competitive ARI scores compared to competing methods (7 out of 12), while the second-place method, GraphST, attained the highest ARI values in 2 samples.

Table S2. The ARI results for each sample from the Coronal Mouse Brain dataset.

Sample	MGGNN	GraphST	SpatialPCA	SpaGCN	BayesSpace	stLearn
control	**0.66987**	0.25039	0.41911	0.46818	−0.00238	0.48982
heme_0030	**0.66455**	0.21862	0.32043	0.28532	−0.00054	0.38018
heme_0125	**0.67653**	0.27204	0.35800	0.26984	0.00096	0.35816
heme_0500	**0.54122**	0.30785	0.30464	0.37575	0.00422	0.50290
heme_1000	**0.70190**	0.33976	0.16942	0.47187	−0.00252	0.59642
hemeHpx_1000	**0.63481**	0.3759	0.42701	0.39410	−0.00247	0.59173
sham	**0.73260**	0.2990	0.30829	0.29014	0.00076	0.46636

This is a quantitative demonstration of Figure 4b. The highest ARI values are listed in bold in the table. MGGNN outperforms all competing methods on this dataset with the help of reliable marker genes.
